# Identification of a novel B-cell epitope in the spike protein of porcine epidemic diarrhea virus

**DOI:** 10.1186/s12985-020-01305-1

**Published:** 2020-04-03

**Authors:** Ning Kong, Qiong Meng, Yajuan Jiao, Yongguang Wu, Yewen Zuo, Hua Wang, Dage Sun, Sujie Dong, Huanjie Zhai, Wu Tong, Hao Zheng, Hai Yu, Guangzhi Tong, Yongjie Xu, Tongling Shan

**Affiliations:** 1 Shanghai Veterinary Research Institute, Chinese Academy of Agricultural Sciences, Shanghai, People’s Republic of China; 2 Jiangsu Co-Innovation Center for the Prevention and Control of Important Animal Infectious Disease and Zoonosis, Yangzhou University, Yangzhou, People’s Republic of China; 3 College of Life Science, Xinyang Normal University, Xinyang, China

**Keywords:** PEDV, Spike protein, Monoclonal antibody, Epitope

## Abstract

**Background:**

Porcine epidemic diarrhea virus (PEDV) infection causes an acute enteric tract infectious disease characterized by vomiting, anorexia, dehydration, weight loss and high mortality in neonatal piglets. During PEDV infection, the spike protein (S) is a major virion structural protein interacting with receptors and inducing neutralizing antibodies. However, the neutralizing B-cell epitopes within PEDV S protein have not been well studied.

**Methods:**

To accurately identify the important immunodominant region of S1, the purified truncated S1 proteins (SA, SB, SC, SD and SE) were used to immunize BALB/c mice to prepare polyclonal antibodies. The antisera titers were determined by indirect ELISA, western blot and IFA after four immunizations to find the important immunodominant region of S1, and then purified the immunodominant region of S1 protein and immunized mice to generate the special antibodies, and then used recombinant peptides to determine the B-cell epitopes of monoclonal antibodies.

**Results:**

Five antisera of recombinant proteins of the spike protein region of PEDV were generated and we found that only the polyclonal antibody against part of the S1 region (signed as SE protein, residues 666–789) could recognize the native PEDV. Purified SE protein was used to immunize BALB/c mice and generate mAb 2E10. Pepscan of the SE protein demonstrated that SE16 (^**722**^SSTFNSTREL^**731**^) is the minimal linear epitope required for reactivity with the mAb 2E10. Further investigation indicated that the epitope SE16 was localized on the surface of PEDV S protein in the 3D structure.

**Conclusions:**

A mAb 2E10 that is specifically bound to PEDV was generated and identified a specific linear B-cell epitope (SE16, ^**722**^SSTFNSTREL^**731**^) of the mAb. The epitope region of PEDV S1 localized in the different regions in comparison with the earlier identified epitopes. These findings enhance the understanding of the PEDV spike protein structure for vaccine design and provide a potential use for developing diagnostic methods to detect PEDV.

## Background

Porcine epidemic diarrhea (PED) is an acute enteric tract infectious disease characterized by vomiting, anorexia, dehydration, weight loss and high mortality in neonatal piglets [1, 2]. The disease was reported in European and Asian pig industries over the last 30 years, with the virus firstly appearing in England and Belgium in the early 1970s [3, 4]. Porcine epidemic diarrhea virus (PEDV), although the etiologic agent of PED has become a severe problem in many Asian countries, including China, Korea, Japan and Thailand [5–8]. Since 2010, the virulent PEDV has become prevalent in swine herds and incurred huge economic losses to the swine industry [9–11]. Due to the lack of effective vaccines, PED is still circulating in the worldwide.

PEDV belonging to the genus *Alphacoronavirus*, family *Coronaviridae*, has an approximately 28 kb genome of single-stranded, positive-sense RNA [1]. The PEDV genome encodes two large polyproteins, an accessory protein and four structural proteins. The structural proteins contain glycosylated spike (S), envelope (E), glycosylated membrane (M) and RNA-binding nucleocapsid (N) proteins [12]. The spike gene can be divided into S1 and S2 domains, as in other coronaviruses and it has multiple functions that can interact with cellular receptors and regulating viral entry and containing neutralizing epitopes to induce neutralizing antibodies [13, 14].

In the present study, we expressed and purified the recombinant truncated PEDV S1 constructs (SA-SE) to immunize BALB/c mice and found that SE, one of the S1 construct (residues 666–789), was the immunodominant region of S1 protein. Furthermore, we utilized the SE protein to immunize BALB/c mice and obtained one SE specific mAb, 2E10. A novel linear B-cell epitope, (^**722**^SSTFNSTREL^**731**^), was subsequently identified using the SE specific mAb 2E10. These results provide valuable information for virus diagnosis and vaccine design.

## Methods

### Cell lines, viruses and plasmids

African green monkey kidney cells (Vero E6) and SP2/0 myeloma cells were cultured in a humidified 5% CO_2_ atmosphere at 37 °C. All the culture media were Dulbecco’s modified Eagle’s medium (DMEM, Hyclone) supplemented with 10% fetal bovine serum (FBS) and antibiotics (0.1 mg/ml of streptomycin and 100 IU/ml of penicillin). PEDV strain JS-2013 was obtained from the Shanghai Veterinary Research Institute (CAAS, China). Plasmid DNA (pCold-TF) containing the S1 gene (1–2367 bp) of PEDV strain JS-2013 was constructed by our own laboratory.

### Expression of the truncated PEDV S1 proteins

The five overlapping fragments, comprising partial length of PEDV S1 gene, were constructed and designated as SA, SB, SC, SD and SE. A *BamH I* site and sixteen extra bases that were homologous to the terminal sequence of the vector were added to the 5′ end. The sequences of the primers used for amplification of the gene in this study are shown in Table 1. All the recombinant plasmids were constructed by ClonExpress II One Step Cloning Kit (Vazyme Biotech, C112–02), according to the manufacturer’s instructions. The truncated segments SA-SE were cloned into pCold-TF vector and the confirmed recombinant plasmids were transformed into *E. coli* BL21 and induced by isopropyl-β-D-thiogalactoside (IPTG) at 16 °C for 24 h. The truncated PEDV S1 proteins were analyzed by sodium dodecylsulfate-polyacrylamide gel electrophoresis (SDS-PAGE) and western blot. All the recombinant proteins were purified by using Nickel Magnetic Beads (Biotool, Shanghai, China) to prepare polyclonal antibodies.

**Table 1:** Sequence of the primers used in this study

Names	Primer sequences (5′ → 3′)	Position^a^
SA	F: **TACCCTCGAG**GGATCCATGAAGTCTTTAACCTACTTCTGGTTGT R: **GCTTGAATTC**GGATCCTTAAGCACAACCTCCACTGTTGTAACA	1–609
SB	F: **TACCCTCGAG**GGATCCATGACATGGGATAATGATCGTGTCACT R: **GCTTGAATTC**GGATCCTTATAAGTGAGGATCTGAGGAATTACTGC	499–1071
SC	F:**TACCCTCGAG**GGATCCATGAATATTAATGACACCTCTCTCATTCTTG R: **GCTTGAATTC**GGATCCTTAAAACCCATTGATAGTAGTGTCAGATG	964–1617
SD	F: **TACCCTCGAG**GGATCCATGCCATCATTTAATGATCATTCTTTTGTT R: **GCTTGAATTC**GGATCCTTACACTATATCATCATCAACATATGCAGC	1513–2142
SE	F: **TACCCTCGAG**GGATCCATGAATTCTAGCTTTTTGGCAGGTG R: **GCTTGAATTC**GGATCCTTAACTAAAGTTGGTGGGAATACTAATATTC	1999–2367
SE1	F: **TACCCTCGAG**GGATCCATGAATTCTAGCTTTTTGGCAGGTG R: **GCTTGAATTC**GGATCCTTAATCATCAACATATGCAGCCTGCTCT	1999–2133
SE2	F: **TACCCTCGAG**GGATCCATGACTAGTGGTGCTGTTTATTCTGTTA R: **GCTTGAATTC**GGATCCTTAGTAGAAGAAACCAGGCAACTCCCTA	2068–2208
SE3	F: **TACCCTCGAG**GGATCCATGGCATATGTTGATGATGATATAGTGG R: **GCTTGAATTC**GGATCCTTAACTGCCAGATTTACAAACACCTATG	2119–2283
SE4	F: **TACCCTCGAG**GGATCCATGTCTAATTGTACAGAGCCTGTGTTGG R: **GCTTGAATTC**GGATCCTTAACTAAAGTTGGTGGGAATACTAATATTC	2224–2367
SE5	F: **TACCCTCGAG**GGATCCATGGCATATGTTGATGATGATATAGTGG R: **GCTTGAATTC**GGATCCTTAGTTAAAAGTGGAGCTAGACAAACTA	2119–2178
SE6	F: **TACCCTCGAG**GGATCCATGATTTCTAGTTTGTCTAGCTCCACTT R: **GCTTGAATTC**GGATCCTTAATTTCTAGTTTGTCTAGCTCCACTT	2149–2208

The introduced restriction enzyme sites *BamH I* are underlined. At 5′ terminal of each sense strand, there is a sequence of TACCCTCGAG (bold) which is as same as the ends of carrier Pcold-TF. At 3′ terminal of each reverse sense strand, there is a sequence of GCTTGAATTC (bold) complement with the ends of carrier Pcold-TF

^a^Location of the synthesized peptides is based on the sequence of S protein of PEDV strain HeB/TS/2016/325b (GenBank accession no. KX907110.1)

### Identification of immunoactivity of truncated proteins

Groups of five 8-week-old female BALB/c mice were intraperitoneally immunized with 50 μg purified truncated PEDV S1 proteins. Antigens were emulsified in the same volume of complete Freund’s adjuvant (Sigma, USA) for the initial immunization, then emulsified in incomplete Freund’s adjuvant on subsequent immunizations at 2-week intervals for 6 weeks. Phosphate-buffered saline (PBS) was used for the controlled trial with the same procedures. Three days after the final boosting, the mice were narcotized and their blood samples were collected from the caudal vein. The collected antisera were diluted 1000-fold and used for indirect ELISA, western blot and IFA to detect the immunoactivity of truncated proteins.

### Development of monoclonal antibody targeting PEDV

Female 8-week-old BALB/c mice were immunized with 50 μg purified protein emulsified in the same volume of complete Freund’s adjuvant via intraperitoneal injection. This procedure was followed by three additional injections at 2-week intervals with the same dose of antigen emulsified in incomplete Freund’s adjuvant. Three days after the final injection, spleen cells from immunized mice were fused with SP2/0 myeloma cells using polyethylene glycol 1450 (PEG1450, Sigma, USA), as previously described [15]. Then the hybridoma cells were seeded into 96-well plates and selected in hypoxanthine-aminopterin-thymidine (HAT) medium and hypoxanthine-thymidine (HT) medium. The cell culture supernatants of surviving clones were determined by indirect ELISA for antibody reactivity and specificity. Positive hybridomas were cloned four times by limiting dilution. Ascites fluids were produced in pristane induced BALB/c mice.

### Indirect enzyme-linked immunosorbent assay (ELISA)

Indirect ELISA was used to identify the immune reactivity of the truncated proteins and the screen of positive hybridoma cells. The ELISA plates were plated with purified PEDV S1 protein or synthesized peptides (400 ng/well) in carbonate bicarbonate buffer (15 mM Na_2_CO_3_, 35 mM NaHCO_3_ [pH 9.6]) and coated at 4 °C overnight. The plates were blocked for 1 h at 37 °C using 5% non-fat dry milk in phosphate buffer with 0.05% Tween-20 (PBST). After being washed thrice, the plates were incubated with 100 μL diluted anti-sera or antibodies at 37 °C for 1 h. The plates were incubated with horseradish peroxidase (HRP) -conjugated goat anti-mouse IgG (Proteintech Group, China) with 1:20,000 dilution in PBST at 37 °C for 1 h after being washed thrice in PBST. Then, plates were washed with PBST and incubated with 50 μL/well of TMB liquid (Amresco, Solon, Ohio, USA) for 15 min at room temperature with protection from light. The results were read with OD450 values after being stopped by 2 M H_2_SO_4_ (50 μL/well).

### Virus infection and western blot analysis

To analyze the anti-sera or antibodies specificity interacted with PEDV, Vero cells were infected with PEDV (multiplicity of infection, MOI = 1), or mock infected with the medium, and then incubated for indicated times as previously described [16]. The cells were harvested and lysed using RIPA lysis buffer (Thermo, USA) containing protease inhibitor cocktail (Bimake, USA) and phosphatase inhibitor cocktail (Bimake, USA) on ice for 5 min. The cell lysates were then separated by 10% SDS-PAGE and transferred to the nitrocellulose (NC) membrane (GE Healthcare, USA). The membranes were blocked with 5% non-fat dry milk in TBST (TBS with 0.1% Polysorbate-20) for 1 h at room temperature (RT). The membranes were subsequently incubated with HRP-conjugated goat anti-mouse IgG (1:6000 dilution in TBST) for 1 h at RT. Proteins were visualized by using SuperSignal West Pico chemiluminescent substrate (Thermo Fisher Scientific, USA) according to the manufacturer’s instructions.

### Indirect immunofluorescence assay (IFA)

Vero cells were plated in a six-well plate and infected with PEDV when the cells reached approximately 90% confluence. At 24 h postinfection, the cells were fixed with 4% paraformaldehyde (Sigma-Aldrich) for 15 min and permeabilized with 0.1% Triton X-100 (Sigma-Aldrich) for 10 min at room temperature. After being washed three times in phosphate-buffered saline (PBS), the cells were blocked with 10% bovine serum albumin (BSA) in PBS for 1 h at 37 °C and then incubated with the primary antibody for 1 h. After three washes with PBS, cells were incubated with Alexa Fluor 488 donkey anti-mouse IgG (H + L) antibody in the dark for 1 h at 37 °C. Following several washes, the fluorescence was visualized by using an Olympus® IX73 inverted microscope.

### Bioinformatics analysis

The spatial position of the identified epitope was analyzed by mapping the location on the 3D structure model of PEDV S [17] by using PyMOL software [18, 19], and the secondary structure of amino acid sequences of the identified epitope was also analyzed by PROTEAN software (DNASTAR’s Lasergene, Inc., Madison, WI, USA) [20].

### Statistical analysis

All results are representative of three independent experiments. Statistical analysis was performed using Prism 5.0 software (GraphPad). Significance was determined by two-tailed Student’s *t* test. Statistical significance: *p < 0.05, **p < 0.001.

## Results

### Immunodominant region of S1 protein

**Fig. 1:**
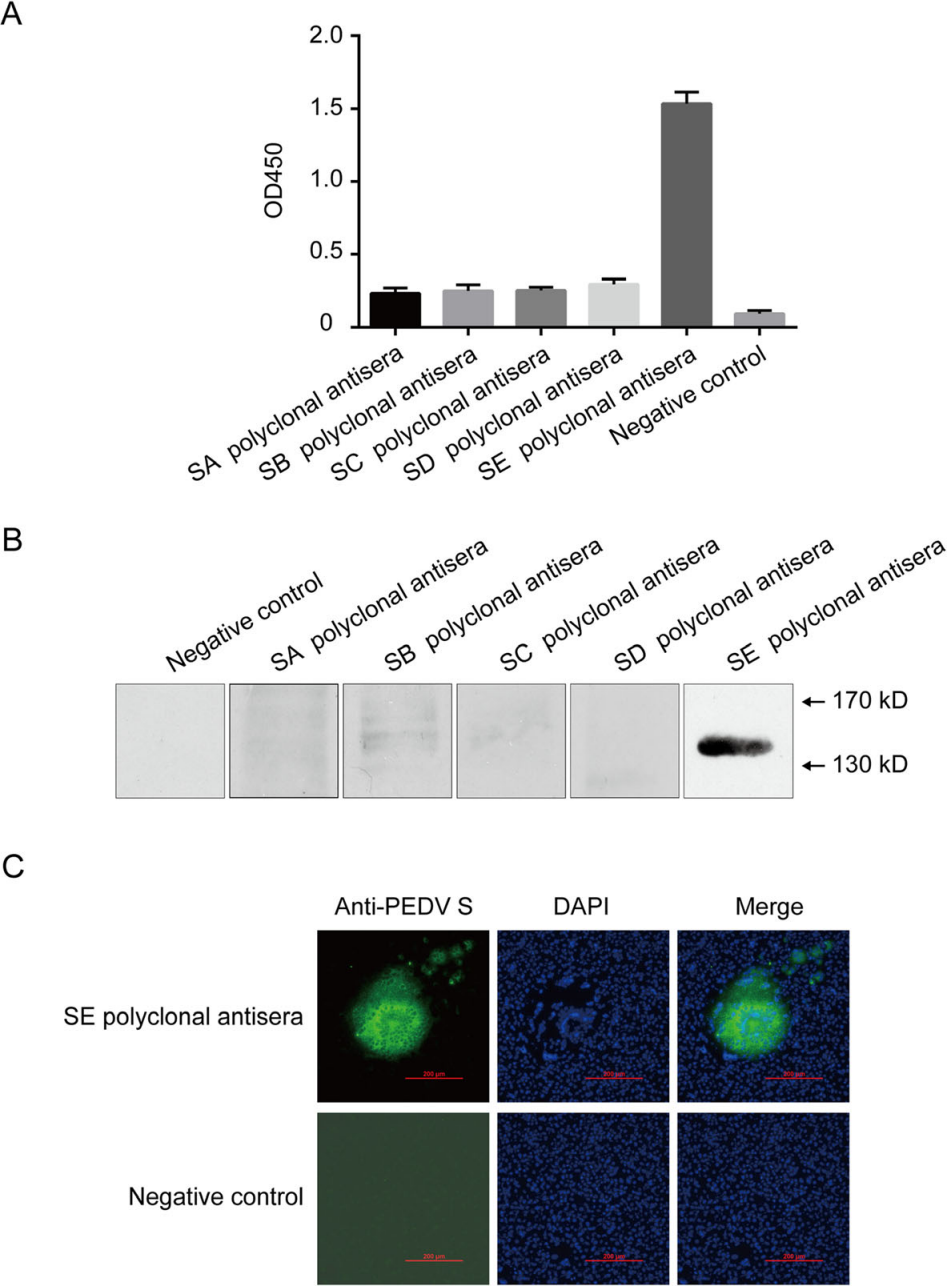
Detection of recombinant proteins’ antigenicity for polyclonal antisera (PcAbs) by indirect ELISA, western blot and IFA. **a** Reactivity of antisera against the recombinant S1 protein by indirect ELISA. **b** Western blot analysis of the recombinant S1 protein with PcAbs. The recombinant S1 protein (approximately 140 kDa) was transferred to the nitrocellulose (NC) membrane, followed by the different PcAbs (negative control, SA polyclonal antisera, SB polyclonal antisera, SC polyclonal antisera, SD polyclonal antisera and SE polyclonal antisera) as primary antibody. **c** Immunofluorescence analysis of SE polyclonal antisera against PEDV. Vero cells were plated in six-well plates and inoculated with PEDV (0.01 MOI). Twenty-four hours later, cells were fixed and incubated with SE polyclonal antisera or normal mouse serum (negative control), and then incubated with Alexa Fluor 488 donkey anti-mouse IgG (H + L) antibody. Scale bars: 200 μm

To accurately identify the immunodominant region of S1, the purified truncated S1 proteins (SA, SB, SC, SD and SE) were used to immunize BALB/c mice to prepare polyclonal antibodies. The antisera titers were determined by indirect ELISA, western blot and IFA after four immunizations. The indirect ELISA and western blot analysis showed that the SE polyclonal antisera had the highest antibody titers against PEDV S1 protein (Fig. 1a and b), suggesting that SE protein was the important immunodominant region of S1. The immunofluorescence signal for the PEDV S1 protein was also detected by the SE polyclonal antisera (Fig. 1c), further confirming that SE polyclonal antisera could recognize the native PEDV. Collectively, these results indicated that the region of SE (666–789 aa) was the immunodominant region of S1 protein.

### Production and characterization of SE protein-specific mAb

**Fig. 2:**
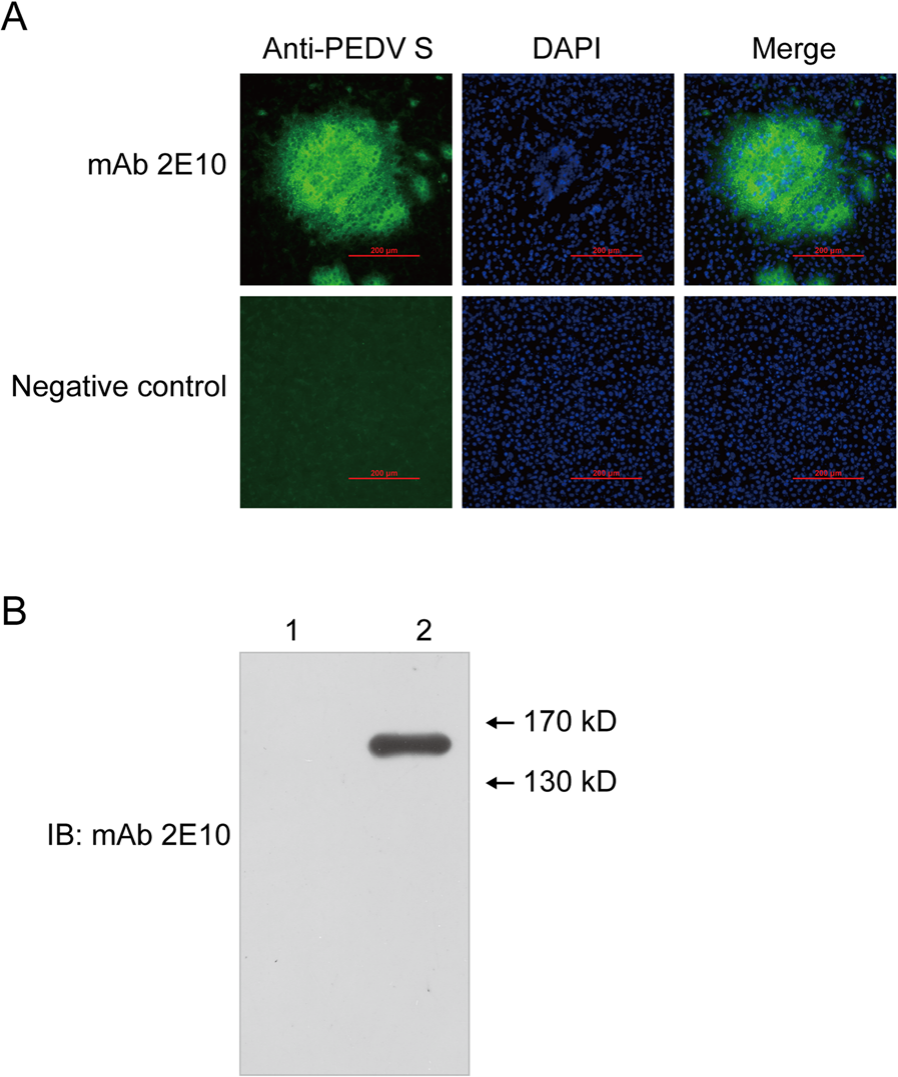
The mAb 2E10 recognizes native S protein of PEDV in PEDV-infected Vero cells. **a** Indirect immunofluorescence assays analyzed the specificity of prepared mAb 2E10 against PEDV. Scale bars: 200 μm. **b** Western blot assays analyzed the specificity of prepared mAb 2E10 against PEDV (S protein approximately 160 kDa). Lanes (1–2): Vero cells, PEDV-infected Vero cells

Purified SE protein was used to immunize BALB/c mice to prepare mAbs, and then determined the antisera titers using indirect ELISA after four immunizations. The mouse with the highest antibody titers against SE protein was used for cell fusion. After being subcloned by limiting dilution and screening for four times, one positive mAb against SE protein was identified and named 2E10. The mAb 2E10 cell clone was used to prepare ascites containing mAbs. The ascites was collected and purified using Nab Protein G Spin Columns (Thermo Fisher Scientific, Rockford, IL). Western blot analysis (Fig. 2a) and immunofluorescence assay (IFA) (Fig. 2b) were used to identify the specificity of the mAbs against PEDV. The results suggested that the mAb 2E10 could be specifically reacted with native PEDV protein.

### Analysis of the immunodominant region of SE with mAb

**Fig. 3:**
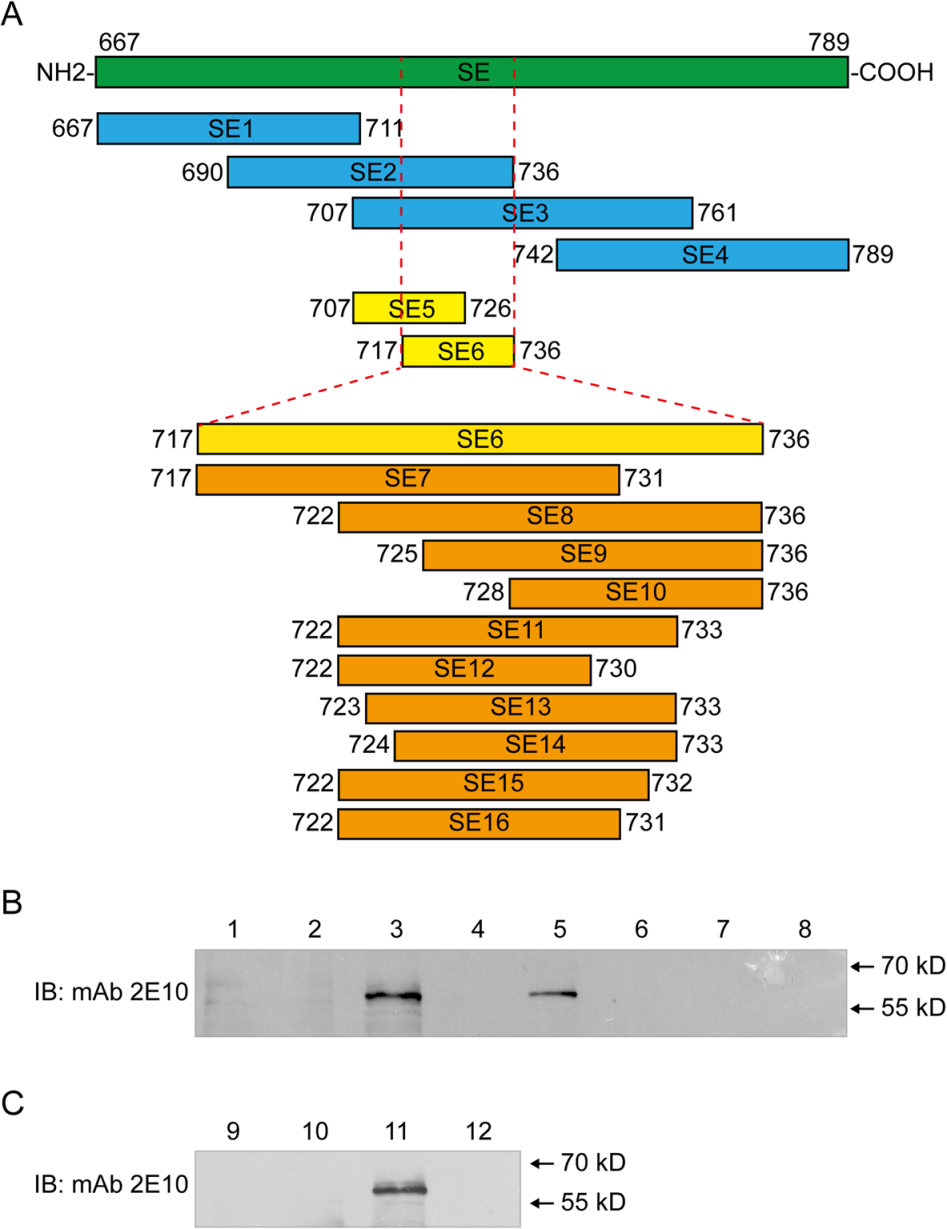
Analysis of the immunodominant region of SE with mAb 2E10. **a** Schematic diagram of the relative locations of the truncated fragments of the SE protein. The boxes represent the truncated SE proteins. The numbers represent the amino acid positions of the SE protein. **b** Identification of SE1, SE2, SE3 and SE4 recognized by mAb 2E10. Lanes: (1–8) expression and non-expression of SE1-His, expression and non-expression of SE2-His, expression and non-expression of SE3-His, expression and non-expression of SE4-His, respectively. **c** Identification of SE5 and SE6 recognized by mAb 2E10. Lanes: (9–12) expression and non-expression of SE5-His, expression and non-expression of SE6-His, respectively

To identify the antigenic epitope recognized by mAb 2E10, four truncated and overlapping 6 × His-tagged peptides (SE1-SE4) spanning the SE fragments were designed (Fig. 3a) and expressed using the bacterial system. The sequences of the primers were also shown in Table 1. All fusion proteins were predominantly expressed in soluble form in bacterial cells. Subsequently, western blot was used to determine immune reactivity between the Mab 2E10 and these SE fragments. The results showed that MAb 2E10 reacted with fragments spanning aa 690 to 736 (SE2) and 707 to 761 (SE3) but not aa 667 to 711 (SE1) or 742 to 798 (SE4), suggesting that the epitope recognized by 2E10 was located in aa 707 to 736 of PEDV S1 protein (Fig. 3b). Furthermore, we generated two deletion constructs of SE (SE5, SE6) that span the overlaps of SE2 and SE3 (Fig. 3a) to identify the antigenic epitope. As demonstrated by western blot, SE6 was recognized by mAb 2E10 (Fig. 3c), which suggested that SE6 (^717^ISSLSSSTFNSTRELPGFFY^736^) epitope may be a harbored antigenic epitope.

### Minimization of the epitope SE6 with mAb 2E10

**Fig. 4:**
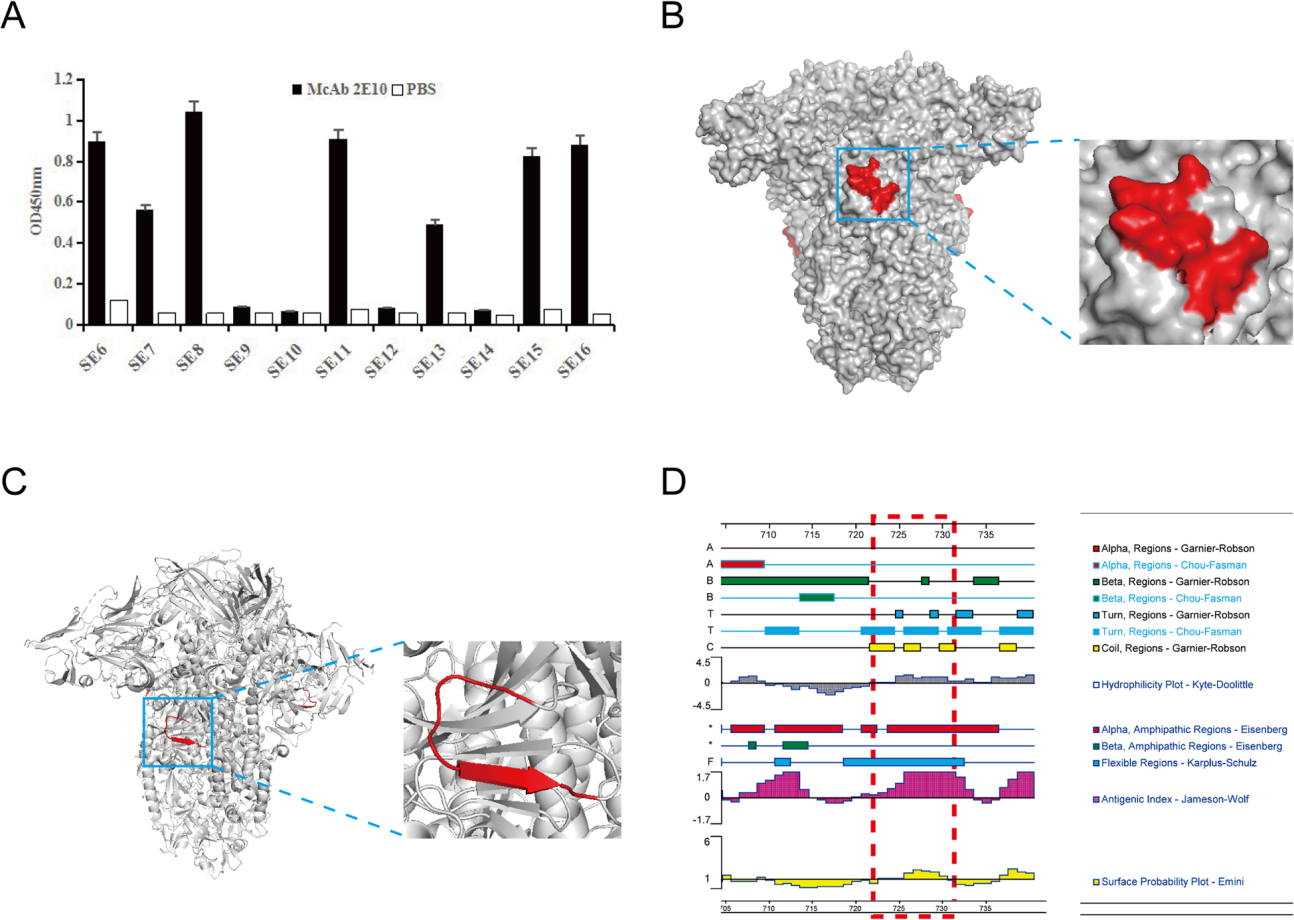
Pepscan of the epitopes SE6 and localization of the epitopes recognized by the mAb 2E10. **a** ELISA analysis of the truncated peptides SE6-SE16 with mAb 2E10 and PBS. The surface **b** and cartoon **c** from SE16 was labeled in the sequence chain view picture of PEDV S, obtained from the Protein Data Bank (PDB, ID: 6U7K). **d** The structural features of SE16 were predicted by PROTEAN software. The epitope SE16 was shown in the boxes

In order to further minimize the epitope of SE6, two shortened peptides (SE7-SE8) were synthesized via solid-phase peptide synthesis (Table 2). Using indirect ELISA, we found that SE8 showed a strong reaction with mAb 2E10 as SE6 did (Fig. 4a). The result suggested that SE8 was the essential region for recognition by mAb 2E10. According to the results, four shortened peptides (SE9-SE12) by deleting three amino acids at either the amino or the carboxy terminus in sequence from the SE8 were synthesized (Table 2). In ELISA, only SE11 could be recognized by mAb 2E10, which indicates that the 2E10-specific epitope was ^**722**^SSTFNSTRELPG^**733**^ (Fig. 4a). With the same method, the other four shortened peptides (SE13-SE16) were synthesized according to the peptide sequence of SE8. The results showed that the peptide SE16 (^**722**^SSTFNSTREL^**731**^) was strongly recognized by the mAb 2E10 (Fig. 4a). Taken together, these results demonstrate that SE16 (^**722**^SSTFNSTREL^**731**^) is the minimal linear epitope required for reaction with the mAb 2E10.

**Table 2:** Sequence of the synthesized peptides corresponding to the epitope SE6

Names	Amino acids sequences and location on S1 protein^a^
SE7	^**717**^ISSLSSSTFNSTREL^**731**^
SE8	^**722**^SSTFNSTRELPGFFY^**736**^
SE9	^**725**^FNSTRELPGFFY^**736**^
SE10	^**728**^TRELPGFFY^**736**^
SE11	^**722**^SSTFNSTRELPG^**733**^
SE12	^**722**^SSTFNSTRE^**730**^
SE13	^**723**^STFNSTRELPG^**733**^
SE14	^**724**^TFNSTRELPG^**733**^
SE15	^**722**^SSTFNSTRELP^**732**^
SE16	^**722**^SSTFNSTREL^**731**^
SE6	^**717**^ISSLSSSTFNSTRELPGFFY^**736**^

^a^Location of the synthesized peptides is based on the sequence of S protein of PEDV strain HeB/TS/2016/325b (GenBank accession no. KX907110.1)

### Conservation and spatial distribution of the novel epitope

To localize the identified epitope SE16, a 3D structural model of PEDV S was obtained from the Protein Data Bank (PDB, ID: 6U7K), and the spatial distribution was analyzed by PyMOL software. The structural visualization revealed that the identified epitope recognized by 2E10 was exposed on the surface of PEDV S structure (Fig. 4b) and partial peptide formed a beta-sheet structure (Fig. 4c). Moreover, the identified epitope SE16 had high antigenic index and hydrophilicity (Fig. 4d). It was suggested that the epitope can easily explore and induce host immune response in those infected animals.

## Discussion

PEDV S protein, one of the most important glycoproteins, contains multiple neutralizing epitopes to induce neutralizing antibodies. The S protein is divided into S1 (residues 1–789) and S2 (residues 790–1383) domains which are defined by the conserved nonamer and the GxCx motifs in coronavirus group II members [21]. Based on the information of other coronaviruses, we found that the S1 domain is thought to contain multiple virus neutralization epitopes and receptor binding-domains [22]. The S2 domain forms the trans-membrane structure of the S protein, but can not induce neutralizing antibodies [23]. These properties make it possible that the S1 could be a suitable candidate for screening and identifying antigenic epitopes. Epitopes are important antigenic elements of virus structural proteins, which could induce antibody production and cell-mediated immunity against viruses. Therefore, epitopes are essential to develop epitope-based vaccines and diagnostics.

Preparation of mAb is required to identify the epitopes of the S1 protein. We had tried to use intact S1 protein to immunize mice, but it was unsuccessful to get the mice serum with immune activity. Because of unidentified but complex factors, we chose to divide the S1 protein into five fragments (SA, SB, SC, SD, SE). The five truncated proteins were expressed and the immunogenicity of the proteins was identified. These results indicated that the SE protein (666–789 aa) had good reactivity as the immunodominant region of S1 protein. Then the SE protein was selected as an immunogen to elicit the formation of monoclonal antibody. After cell fusion and four times of selection, mAb 2E10 was chosen because of its specific reactivity with the SE protein as well as the native S protein PEDV.

Several domains containing neutralizing epitopes within the S protein were identified, such as residues 499–638 [24], residues 636–798 [25], residues 592–607 [26] and residues 575–639 [27]. These mocking epitopes had antigenic similarities with the PEDV neutralizing epitopes. In this study, we expressed a series of truncated proteins (SE1-SE6) to map the epitopes of SE protein. The SE6 protein could be recognized by mAb 2E10. Generally, linear epitopes consist of six to nine or more continuous amino acid residues. So, the pepscan method was used to truncate the epitope SE6 by deleting three amino acids at either the amino or the carboxy terminus in sequence respectively. A total of ten sub-segments (SE7-SE16) was synthesized and the results of ELISA suggested that SE16 could react with mAb 2E10. Above all these results, the epitope SE16 is the immunodominant region of PEDV S protein. The epitope SE16 (^722^SSTFNSTREL^731^) was highly conserved among different strains of virulent PEDV. Further, the 3D structural visualization showed that the epitope SE16 presented on the surface of PEDV S and had a feature of high antigenic index and hydrophilicity. The character of this location makes this epitope easily explore and induce host immune response in the infected animals.

## Conclusions

We prepared a mAb 2E10 that is specifically bound to PEDV and identified a specific linear B-cell epitope (SE16, ^**722**^SSTFNSTREL^**731**^) of the mAb. The epitope region of PEDV S1 localized in the different regions in comparison with the earlier identified epitopes. Therefore, the identified region is a novel B-cell antigenic epitope region of PEDV S protein and the identified epitope has potential use for developing diagnostic reagent and effective vaccines for PEDV.

## Data Availability

Data generated and analyzed in this study are presented in this manuscript. Data can be obtained by contacting the corresponding author.

## Abbreviations

ELISA: Indirect enzyme-linked immunosorbent assay
HAT: Hypoxanthine-aminopterin-thymidine
HT: Medium and hypoxanthine-thymidine
IFA: Indirect immunofluorescence assay
IPTG: Isopropyl-β-D-thiogalactoside
mAb: Monoclonal antibody
PBS: Phosphate-buffered saline
PED: Porcine epidemic diarrhea
PEDV: Porcine epidemic diarrhea virus
RT: Room temperature
SDS-PAGE: Sodium dodecylsulfate-polyacrylamide gel electrophoresis
TCID_50_: 50% tissue culture infectious doses
